# SARS-CoV-2 in Atmospheric Particulate Matter: An Experimental Survey in the Province of Venice in Northern Italy

**DOI:** 10.3390/ijerph19159462

**Published:** 2022-08-02

**Authors:** Alberto Pivato, Gianni Formenton, Francesco Di Maria, Tatjana Baldovin, Irene Amoruso, Tiziano Bonato, Pamela Mancini, Giusy Bonanno Ferraro, Carolina Veneri, Marcello Iaconelli, Lucia Bonadonna, Teresa Vicenza, Giuseppina La Rosa, Elisabetta Suffredini

**Affiliations:** 1 Department of Civil, Environmental and Architectural Engineering (ICEA), University of Padua, 35131 Padova, Italy; alberto.pivato@unipd.it; 2 Environmental Agency of Veneto Region (ARPAV), 30171 Mestre, Italy; gianni.formenton@arpa.veneto.it; 3 LAR Laboratory, Dipartimento di Ingegneria, University of Perugia, 06125 Perugia, Italy; francesco.dimaria@unipg.it; 4 Hygiene and Public Health Unit, Department of Cardiac, Thoracic, Vascular Sciences and Public Health, University of Padua, 35131 Padova, Italy; irene.amoruso@unipd.it; 5 Società Estense Servizi Ambientali (S.E.S.A. S.p.A.), 35042 Este, Italy; tizianobonato7@gmail.com; 6 Department of Environment and Health, Istituto Superiore di Sanità, 00161 Rome, Italy; pamela.mancini@iss.it (P.M.); giusy.bonannoferraro@iss.it (G.B.F.); carolina.veneri@guest.iss.it (C.V.); marcello.iaconelli@iss.it (M.I.); lucia.bonadonna@iss.it (L.B.); giuseppina.larosa@iss.it (G.L.R.); 7 Department of Food Safety, Nutrition and Veterinary Public Health, Istituto Superiore di Sanità, 00161 Rome, Italy; teresa.vicenza@iss.it (T.V.); elisabetta.suffredini@iss.it (E.S.)

**Keywords:** airborne transmission, COVID-19, particulate matter, SARS-CoV-2 in air, transport carrier, air pollution

## Abstract

Analysis of atmospheric particulate matter (PM) has been proposed for the environmental surveillance of SARS-CoV-2. The aim of this study was to increase the current knowledge about the occurrence of SARS-CoV-2 in atmospheric PM, introduce a dedicated sampling method, and perform a simultaneous assessment of human seasonal coronavirus 229E. Thirty-two PM samples were collected on quartz fiber filters and six on Teflon using a low- and high-volumetric rate sampler, respectively, adopting a novel procedure for optimized virus detection. Sampling was performed at different sites in the Venice area (Italy) between 21 February and 8 March 2020 (*n* = 16) and between 27 October and 25 November 2020 (*n* = 22). A total of 14 samples were positive for Coronavirus 229E, 11 of which were collected in October–November 2020 (11/22; positivity rate 50%) and 3 in February–March 2020 (3/16 samples, 19%). A total of 24 samples (63%) were positive for SARS-CoV-2. Most of the positive filters were collected in October–November 2020 (19/22; positivity rate, 86%), whereas the remaining five were collected in February–March 2020 at two distinct sites (5/16, 31%). These findings suggest that outdoor PM analysis could be a promising tool for environmental surveillance. The results report a low concentration of SARS-CoV-2 in outdoor air, supporting a scarce contribution to the spread of infection.

## 1. Introduction

COVID-19 is a viral respiratory infectious disease caused by novel SARS-CoV-2. Its relevant route of transmission is via droplets (>5–10 μm) and aerosols (≤5 μm) exhaled by infected individuals upon breathing, speaking, coughing, and sneezing.

In the early phases of the pandemic, a promising line of research started to focus on the detection of viral nucleic acid in non-clinical samples, with the aim of setting up strategies of environmental surveillance complimentary to the swab-based clinical surveillance. The main expectations for such approaches consisted of their versatile role in community monitoring [1]. The main environmental surveillance approach is that of wastewater-based epidemiology (WBE), which, even in the pre-COVID-19 era, had been described to investigate peculiar health and lifestyle aspects of given communities, e.g., the use and abuse of both licit and illicit drugs [2,3]. During the COVID-19 pandemic, WBE was successfully exploited to monitor the presence of SARS-CoV-2 in untreated wastewater [4,5,6,7,8]. A recent study also discussed the long-term utility of WBE in the scenario of a SARS-CoV-2-endemic future. Among the forthcoming roles of wastewater surveillance, the monitoring of viral concentrations in wastewater for quantitative estimation of disease incidence, as well as early warning and neighborhood sampling to detect local outbreaks or even clustering at the building level, have been well highlighted. The juxtaposition of WBE and clinical data for cost-efficient population surveillance, as well as metagenomics of wastewater samples, to track variant circulation represents the further potential of WBE [9].

In addition to WBE, the possible mechanisms of airborne transmission for SARS-CoV-2, and hence the exploitability of surveillance systems based on the air environmental matrix have been promptly investigated and discussed, especially with respect to indoor environments [10,11]. However, in an outdoor environment, the abovementioned virus-laden vectors could interact with pre-existing atmospheric particles, thus creating clusters [12,13,14]. Within this framework, such clusters could hypothetically transport the virus long distances [15]. Consequently, from an interdisciplinary perspective, the analysis of outdoor airborne particles has been recently suggested as another useful tool for detection of the presence of SARS-CoV-2 in broad areas [16,17,18].

Two main approaches have been developed for this kind of assessment: a statistical survey focused on the study of the relationship between excess particulate matter (PM) concentration limits and the prevalence of COVID-19 cases detected in the same areas, as well as an experimental survey for the detection of viral RNA in PM samples. Several authors have applied the first approach in many cities in Italy, China, and the United States [19,20,21,22,23,24,25]. The analyzed relationship is not proof of the effective transport capacity of the virus through PM. Most statistical studies rely on the concept that a high concentration of PM favors a “boost” process for the spread COVID-19. In other terms, the populations that live in areas/cities with high levels of pollutants (e.g., several exceedances of PM concentration limits) are subjected to a chronic inflammatory state of the respiratory tract, which makes them more susceptible to respiratory viruses. On the contrary, only a few studies have experimentally evaluated the presence of the etiological agent SARS-CoV-2 in outdoor air PM [8]. Pivato et al. [26], Chirizzi et al. [27], and Setti et al. [14,25,28] applied this approach in urban areas of northern and southern Italy, and Linillos-Pradillo et al. [29] in Madrid (Spain). However, these studies did not reach any conclusive inference on the potential presence or absence of viral RNA in PM, probably due to the different methodologies applied and to the many variables and dynamics influencing the overall process (see Table 1).

It is also worth considering that the molecular detection of SARS-CoV-2 RNA is not informative in terms of the viability and infectivity of the virus; to the best of our knowledge, the latter has not been assessed in any of the studies mentioned. A realistic hypothesis is that in addition to the evident dilution effect, outdoor airborne transmission results are much less probable than the indoor route because the viral integrity is strongly influenced by environmental factors, such as drying processes and sunlight exposure. The question as to whether SARS-CoV-2 is transmitted by particulate air pollution remains a controversial topic in the scientific community [34]. This should be further addressed in the future by specific research activities, which are outside the scope of the current paper.

The aim of the present study was to investigate the potential presence of SARS-CoV-2 and HCoV-229E (i.e., a human seasonal influenza-like illness coronavirus) in a representative series of PM filters collected in the province of Venice (northern Italy) by means of a dedicated experimental activity. HCoV-229E is responsible for common cold symptoms [35] and, as SARS-CoV-2, is thought be of zoonotic origin [36]. In cohort studies, HCoV-229E, as well as other common seasonal coronaviruses (e.g., HCoV-NL63 and OC43), are often detected during the winter season [37,38]. Therefore, HCoV-229E was included in this survey as a suitable endemic-circulating comparison to confirm the detectability of coronaviruses in PM. Additionally, specific operative measures are suggested for the optimization of viral sampling and detection in PM and, consequently, specific instructions for a standard reference method, which is currently lacking, suited for surveillance and early warning purposes to investigate the spatiotemporal characteristics of COVID-19 and other infectious diseases.

To the best of our knowledge, the novelty of this work consists of (i) the extension of experimentation on new particulate samples, considering that international experiences are extremely scarce; (ii) the introduction of optimized sampling methods for viral detection, which have never been tested for SARS-CoV-2; and (iii) a multipathogen survey of respiratory viruses, including HCoV-229E, extendible in the future to other relevant seasonal respiratory viral pathogens (e.g., influenza virus, rhinoviruses, etc.).

## 2. Materials and Methods

### 2.1. Experimental Design and Sampling Strategy

**Figure 1:**
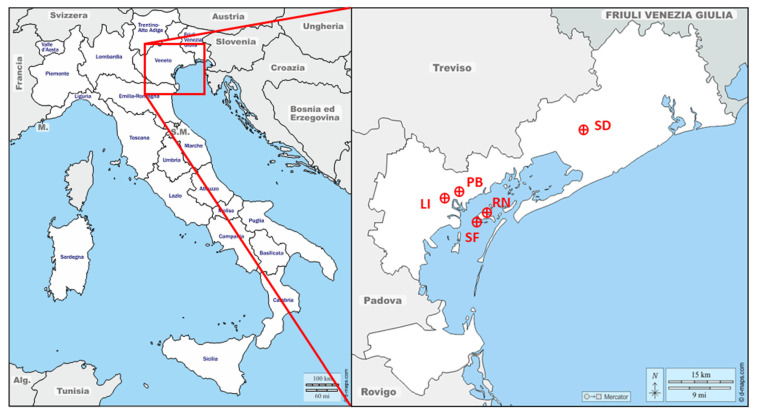
Map of the investigated area. Red dots pinpoint the PM sampling locations in Venice province (VE). LI: Via Lissa; PB: Parco Bissuola; SF: Sacca Fisola; RN: Rio Novo; SD: San Donà (maps from: http://d-maps.com).

Five distinct sites (LI: Via Lissa; RN: Rio Novo; SF: Sacca Fisola; PB: Parco Bissuola; and SD: San Donà) in the province of Venice (northern Italy) (Figure 1 and Table 2) were selected for sampling. Since the initial spread of the pandemic in Italy (February–March 2020), a total of 38 air PM filter samples were collected: 16 samples between 21 February and 8 March 2020 (16 days) and 22 samples between 27 October and 25 November 2020 (29 days). The sampling sites are mapped in Figure 1, with the full details presented in Supplementary Materials S1 (SM1). The sites were classified according to European Directive 2008/50/EC. The average population density in the study areas is 576 inhabitants per square kilometer [39].

**Figure 2:**
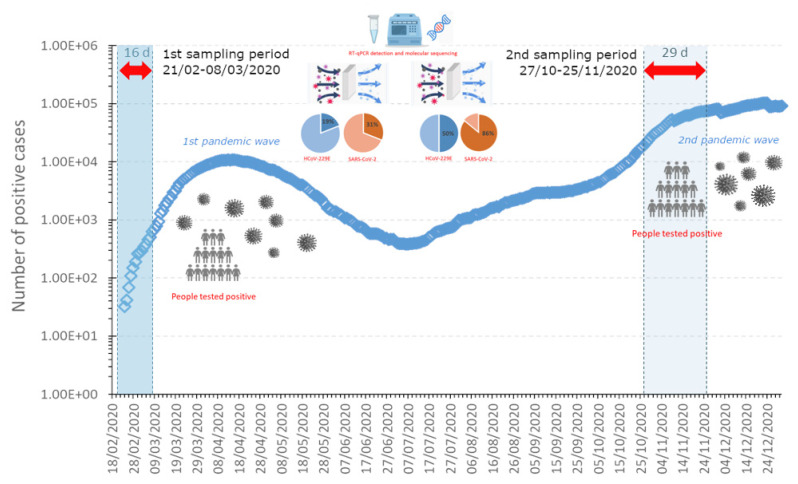
Local epidemic curve of SARS-CoV-2 (i.e., number of daily confirmed COVID-19 cases) for the Veneto region during the two PM sampling periods. First sampling period: 21 February–8 March 2020 (16 days); second sampling period: 27 October–25 November 2020 (29 days).

The evolution of the local SARS-CoV-2 epidemic curve shows, in the first sampling period, an increasing trend from a few to 623 confirmed cases, whereas during the second sampling period, there was a significant increase in COVID-19 cases, i.e., from 19,517 to 75,138 (Figure 2).

Meteorological conditions were registered by the weather station closest to each sampling site, which are also provided in SM1. During the first sampling period, the average daily temperature was 8.24 °C (standard deviation (SD) = 1.89), the average daily wind density was 1.23 m/s (SD = 0.40), and precipitation events with an intensity >10 mm per day were observed only for two samples. In the second period, the average daily temperature was 11.58 °C (SD = 2.50), the average daily wind density was 0.86 m/s (SD = 0.61), and precipitation events with an intensity >10 mm per day were not observed.

Two sampling devices were employed: a low-volumetric rate (LVR; 23–54 m^3^/sample) sampler and a high-volumetric rate sampler (HVR; >250 m^3^/sample). Of 38 samples, 32 were collected using an LVR sampler, in compliance with EN 12341:2014, using quartz fiber filters with a diameter of 47 mm. Six samples were collected in the same sites with the HVR sampler using PTFE, i.e., Teflon, filters with a 3 µm porosity and a diameter of 142 mm. This strategy could not be applied during the first sampling period because at that time, the knowledge on this topic was not adequately developed, and the public health emergency did not allow for new experimental designs.

The LVR sampling approach was previously described in detail by Pivato et al. [26]. It was developed for routine monitoring of air quality in the Veneto region; hence, it is not optimized for virus detection. On the contrary, the HVR approach upgrades the following operative conditions relevant for virus detection:

- Increased sampled air volume: considering that a very low average outdoor concentration of SARS-CoV-2 RNA has been estimated (i.e., <1 genome copy (g.c.)/m^3^) [12,40] and the potential degradation of viral nucleic acid during and after the formation of a virus/PM cluster, the sampled air volume was increased relative to the former method—from 23–54 m^3^/sample to >250 m^3^/sample. This should guarantee the presence of a number of RNA genomic copies above than the limit of detection (LOD) of the molecular assays commonly used for SARS-CoV-2 detection (1–2 g.c./µL).
- Adoption of higher-performance filter typology: in areas such as the investigated sites, characterized by unfavorable atmospheric conditions (i.e., frequent atmospheric stability enhances the age of air mass), Teflon filters have demonstrated improved performance compared to quartz filters for PM collection [41]. Teflon filters are biologically and chemically inert and can meet extreme conditions of chemical compatibility and temperature. Moreover, the wider surface of Teflon filters permits partitioning of the filter into multiple pieces. Consequently, simultaneous analyses can be performed on a single PM sample, such as PM gravimetric estimation, as well as chemical and (micro)biological analysis.
- Adoption a different sample storage modality: LVR samples were retained inside the sampling station for three to four days in containers kept in the dark at 20 °C before reaching the laboratory. Although the LVR method certainly suits PM analysis, for viral sampling and nucleic acid detection, it is recommended that filters are immediately analyzed after sampling or frozen at −20 °C until further processing.

To prevent contamination of samples, the following precautions were taken: technicians handling the filters, regardless of the sampling approach, always wore gloves and a surgical mask.

### 2.2. Filter Processing and Viral RNA Extraction

Before processing, the filters were spiked with a process control virus (mengovirus strain MC_0_) to assess viral recovery. Briefly, 100 µL of the viral suspension was carefully spotted on the side holding the PM, and the filters were left to dry for 30 min in a flow cabinet. The whole quartz fiber filters or a 1/4 section of the larger Teflon filters were then transferred to an extraction tube containing 2 mL of lysis buffer (NucliSENS extraction system, BioMerieux, Marcy l’Etoile, France) for direct extraction of nucleic acids. The sampled were then incubated with lysis buffer for 30 min in an orbital shaker (300 rpm) to allow for uniform overrunning of the buffer on the membranes, followed by centrifugation (10 min at 1400× *g*) into sediment residues. The buffer was then decanted and transferred to a clean tube, and RNA extraction was completed using a MiniMAG semiautomatic platform (BioMerieux, France) according to the manufacturer’s instructions. To ensure the absence of inhibition in real-time RT-PCR detection, the extracted RNAs (100 µL) were further purified using a one-step PCR inhibitor removal kit (Zymo Research, Irvine, CA, USA). The RNAs were stored at −80 °C until molecular testing. A blank filter (unspiked) was also processed with the same protocol as the negative control.

### 2.3. Real-Time RT-qPCR Detection

Recovery of the process control virus was evaluated using real-time RT-PCR, as described elsewhere [42]. Coronavirus 229E was detected with the primers, probe, and conditions described in [30]. SARS-CoV-2 detection and quantification were performed using a real-time RT-qPCR targeting the Orf1b nsp14 region of the SARS-CoV-2 genome, which was developed for detection of the virus in wastewater [30] and previously successfully applied to nasopharyngeal swabs [43], bivalve mollusks [44], and solid waste [1]. The reaction mix (25 µL total volume) included 5 µL of the sample RNA, 1× reaction buffer, 1 µL of the enzyme mix, 1.67 µL of the detection enhancer (all included in the AgPath-ID one-step RT-PCR reagents kit; Life Technologies, Carlsbad, CA, USA), 500 nM of forward primer ID2297, 900 nM of reverse primer ID2298, and 250 nM of probe ID2299 [30]. The amplification conditions were as follows: reverse transcription at 50 °C for 30 min, inactivation at 95 °C for 5 min, and 45 cycles of 15 s at 95 °C and 30 s at 60 °C, with PCR runs performed on a QuantStudio 12K Flex instrument (ThermoFisher Scientific, Waltham, MA, USA). A single reaction was used for process control and coronavirus 229E analysis, whereas, given the expected low concentration of viral RNA, the samples were tested in quadruplicate for SARS-CoV-2. A cycle threshold (Ct) cutoff value of 40 was applied for interpretation of the results. To quantify the viral RNA, a double-strand DNA standard curve (range 1 × 10^1^ to 1 × 10^5^ copies/µL) was constructed using a synthetized fragment (Eurofins Genomics, Ebersberg, Germany) quantified by fluorometric measure (Qubit, ThermoFisher Scientific, USA). PCR inhibition was ruled out by an external amplification control (in vitro synthetized RNA encompassing the nsp14 target region of the real-time PCR). The reagents and environmental contamination were monitored by including two negative controls in each PCR run.

### 2.4. Molecular Characterization

Samples detected as positive by real-time PCR underwent molecular characterization by sequencing the partial spike gene, as described by La Rosa et al. [45], with slight modifications. Briefly, amplification was carried out with three reactions: two short-nested PCRs (≈320 bps, ID_972/973 and ID_974/975) and one long-nested PCR (≈1600 bps, ID_979/980). Reverse transcription and the first cycle were performed using a SuperScript III one-step RT-PCR system (Invitrogen, Waltham, MA, USA) with the following conditions: 45 °C for 20 min and 94 °C for 2 min, followed by 35 cycles of 94 °C for 15 s, 58 °C for 30 s, and 68 °C for either 30 s (PCR ID 972 and 974) or 1 min and 45 s (PCR ID 979), with a final extension at 68 °C for 5 min. Each reaction included a primer concentration of 400 nM and 10 μL of the sample RNA in a final volume of 25 μL. Nested PCR was performed with a 25 μL volume using a Phusion Hot Start II DNA polymerase with GC buffer (ThermoFisher Scientific, USA), 2 μL of the first PCR product, and 400 nM of each primer, with the following cycling conditions: initial denaturation at 98 °C for 30 s followed by 45 cycles of 98 °C for 10 s, 62 °C for 30 s, 72 °C for either 30 s (PCR ID 973 and 975) or 1 min (PCR ID 980), and a final extension at 72 °C for 10 min. Standard precautions were taken to avoid laboratory contamination. The PCR products were observed by gel electrophoresis (1.5% agarose gel, ethidium bromide-stained), purified using a GRS PCR and gel band purification kit (GRISP, Portugal), and sequenced on both strands (Eurofins Genomics, Germany). Mutation analysis was performed using CoVsurver (www.gisaid.org/epiflu-applications/covsurver-mutations-app) (accessed on 3 September 2021). The sequences were submitted to GenBank under the accession numbers OK036454–OK036461.

## 3. Results and Discussion

The detailed analytical results are summarized in Supplementary Materials S2 (SM2). Viral recovery was calculated for all of the 38 tested filters. Three samples (IDs 5, 6, and 36) presented values that were not statistically in accordance with the others. Excluding these samples identified as unreliable outliers, the mean viral recovery in the tested filters was 0.8% (median, 0.61; range, 0.14–4.16%). The average recovery efficiency for the quartz fiber filters was 0.83% (median 0.62%), whereas for the Teflon filters, it was 0.68% (median 0.60%). Among the quartz fiber filters, the recovery efficiency was slightly higher in PM10 than in the PM2.5 samples, i.e., 0.86% and 0.53%, respectively. Teflon filters were used to collect only PM10 samples, meaning a similar comparison is not feasible.

The median recovery obtained in the present study, which is lower than that reported by other authors [25,26,27,29], could be attributed to the analytical approach adopted (i.e., filter spiking with mengovirus, followed by 30 min drying in a flow cabinet) (Table 1). This approach promoting the adhesion of the process control virus to the PM, provides better simulation of the conditions under which viral targets are recovered from filters. PCR inhibition was absent in 35 samples and <10% in three samples.

A total of 14 samples were positive for HCoV-229E, 3 of which were collected in the first sampling period (3/16, positivity rate 19%) and of which were 11 collected during the second sampling period (11/22, positivity rate 50%). The Ct values for the HCoV-229E-positive samples ranged from 33.99 to 38.35. These Ct results, although not quantitative per se, correspond to a theoretical concentration difference of 1.3 log_10_ between the highest and the lowest loaded samples. The obtained results indicate a significant occurrence of HCoV-229E in PM, although associated with a wide variability in its concentrations, which may be related to local peaks of population transmission/circulation.

The SARS-CoV-2 genome was successfully detected in 24 samples; 13 samples were positive in a unique analytical replicate, 8 in 2 replicates, 2 in 3 replicates, and only 1 sample scored positive in all replicates. The viral RNA concentrations estimated for positive samples (average value of 0.25 g.c./μL of RNA, min 0.10–max 0.52) confirmed that most of the analytical replicates were close to the LOD (0.41 g.c./μL of RNA) and significantly lower than the LOQ of the real-time RT-qPCR assay adopted in the study (3.71 g.c./μL of RNA). Therefore, the quantitative results should be considered estimated values. Based on these results, the viral load in the air samples was calculated to be, on average, 0.8 ± 0.5 g.c./m^3^ (min 0.1–max 2.1 g.c./m^3^).

Positive samples were mostly collected between October and November 2020 (19 positive samples out of 22; positivity rate, 86%), whereas only five samples were collected between 28 February and 7 March 2020 (5/16; positivity rate, 31%). The detection of SARS-CoV-2 at the end of February 2020 indicates the early occurrence of the virus in air particulate matter during the initial spread of the pandemic. Interestingly, all of the samples with a viral concentration equal to or above the average were associated with samples collected in October and November 2020 in the same location (Rio Novo, VE). Overall, nine samples were positive both for HCoV-229E and SARS-CoV-2, all of which were collected during the second sampling period, that is, between October and November 2020.

Amplification of the SARS-CoV-2 spike region by conventional nested RT-PCR was successfully achieved in eight samples (one with PCR ID 975, seven with PCR 973, and none with PCR 980). Sequences spanning amino acid regions 58–150 of the spike gene were obtained for four samples (IDs 18, 19, 27, and 32), whereas different sequences were obtained for the other four (sample IDs 24 aa 58 to 125, ID 25 aa 58 to 143, ID 29 aa 76 to 150, and ID 14 aa 523 to 579). In agreement with the expected results for samples collected before December 2020, all sequenced fragments, except for sample ID 32, displayed a 100% identity with the corresponding region of the prototype sequence NC_045512 (Wuhan strain). Sample ID 32, collected on 9 November 2020, displayed two rare mutations of the spike gene (F59L and T114I) for which nucleotide misincorporation during reverse transcription or polymerization cannot be excluded. To the best of our knowledge, this is the first study to achieve confirmation and molecular characterization of SARS-CoV-2 in PM via sequencing.

Thus, the results indicate an increased probability of detecting a coronavirus in the second period, probably due to, at least for SARS-CoV-2, a higher number of infected individuals, as shown in Figure 2. No specific correlation was found with respect to the simultaneous presence of HCoV-229E and SARS-CoV-2, either in terms of the PM concentration or virus detection.

Considering only the period between October and November 2020, during which both of the sampling strategies were applied, the following results were obtained:

- Samples collected using the LVR method (i.e., optimized for PM analysis) (*N* = 16): 8 and 14 samples were positive for HCoV-229E and SARS-CoV-2, respectively;
- Samples collected using the HVR method (i.e., optimized for virus analysis) (*N* = 6): three and five samples were positive for HCoV-229E and SARS-CoV-2, respectively.

It is also worth mentioning that the SARS-CoV-2 concentration in an outdoor atmosphere, when detected, is very low, suggesting a low probability of PM contributing to the spread of infection. Moreover, the survival of SARS-CoV-2 in outdoor conditions and in PM seems quite limited. Consist with these results, the authors of some recent papers have theoretically discussed the possible direct and indirect role of exposure to PM in the COVID-19 pandemic, although still hinting at the need for further empirical investigation [46]. In addition, the infectivity of the captured virus was not analyzed.

Comparing these experimental results with those obtained in previous studies in Italy and Spain, we note different experimental designs, with complete information about the scientific approach of many studies still missing. Therefore, the influence of several factors on the specific results is not clear, such as socioeconomic activity in the investigated area that may result in reduced circulation of the SARS-CoV-2, atmospheric and meteorological conditions, virus degradation during sampling, sampler and sampling material performances, sample pretreatment (included freezing), the effects of inhibitors, etc. [47].

## 4. Conclusions

Overall, 38 PM samples were collected and successfully processed. A total of 14 filters (37%) were positive for HCoV-229E and 24 for SARS-CoV-2 (63%). During the first wave of the COVID-19 pandemic in Italy (28 February to 7 March 2020), a total of 16 samples were collected: three PM filters were positive for HCoV-229E (19%) and five for SARS-CoV-2 (31%). Later, among the 22 samples collected between October and November 2020, 11 were positive for HCoV-229E (50%) and 19 for SARS-CoV-2 (86%). The divergence between the positivity rate of the two sampling periods should be interpreted with consideration of the considerable difference in the prevalence of COVID-19, with the second sampling period showing a higher number of positive cases (see Figure 2).

Moreover, when comparing the results with those of previous studies from Italy and Spain, the detectability of viral genomes seemed dependent on variegated additional factors, including socioeconomic characteristics (presence of industrial activities, residential areas, roads, etc.) of the investigated areas, which ultimately may have resulted in reduced circulation of SARS-CoV-2; atmospheric and meteorological conditions; virus degradation during sampling; sampler and sampling material performance; sample pretreatment (included freezing); the effects of inhibitors; etc. To date, no published study has evaluated the infectivity of recovered viruses.

In addition, with the aim of improving the detection of SARS-CoV-2, as well as other respiratory viruses, in atmospheric PM as a tool for community surveillance, some suggestions can be obtained from the current experimental survey and literature review:

- The adopted methods do not allow for the assessment of SARS-CoV-2 infectivity; therefore, we cannot draw any conclusion in terms of the spread of infection with respect to the possible role of PM in SARS-CoV-2 diffusion. In particular, infectivity assays should be conducted in an appropriate biosafety-level (BSL) facility.
- Large volumes of air should be sampled in consideration of the expected low number of viral particles in environmental samples. The HVR approach is preferred.
- Filters appear to be the most effective devices for the simultaneous capture of micrometric (e.g., smaller) particles and the collection of large air volumes [48].
- Large Teflon filters should are preferable to quartz fiber filters due to their overall better recovery performance and the possibility of being fractioned for multiple analytical purposes, e.g., chemical characterization of PM.
- Shorter sampling phases (i.e., <1–2 h) should be used to ensure a low SARS-CoV-2 degradation rate.
- Standardized procedures and methods for outdoor sampling and detection of airborne viruses require further investigation before they can be established.

Although further field activities and empirical studies are needed to both strengthen and refine the present understanding of the mechanisms affecting the outdoor spread of infection and the possible direct/indirect role of PM as a viral carrier, our findings indicate the promise of analysis of outdoor PM as an additional tool for the environmental epidemiological surveillance of communities. Future research is required to investigate the contribution of the abovementioned factors to transmission dynamics.

Intercalibration and integration of environmental surveillance data with chemistry transport models for PM should also be assessed, as they are thought to share mutual influences [49]. In particular, atmospheric PM transport models have been used for years to predict PM10 concentrations on a regional scale. However, with respect to the transport of active viral particles, it might be more appropriate to develop dispersion models with a shorter range in order to understand whether areas with a high incidence can ultimately act as virus dispersion sources. Nevertheless, as such models were originally developed for particles that do not deteriorate in a short time, it will be necessary to develop viral dispersion models, given the decay time of various viruses in the atmosphere under different conditions of humidity, temperature, irradiation, and air pollution quality [50].

Furthermore, the consolidation of environmental surveillance methods will play a role even after the end of the ongoing COVID-19 pandemic so that, in addition to SARS-CoV-2, virtually any microbial pathogen could be promptly identified and monitored in suitable samples. Moreover, in the short term, broader diffusion of novel approaches, such as LOD-improving digital PCR, metagenomics for direct sequencing and analysis of whole microbial communities, and biosensing, are expected to further enhance the potential of environmental monitoring and surveillance.

## Figures and Tables

**Table 1:** Thorough comparison of the materials and methods used in the current study with those previously proposed by other authors [25,26,27,29].

Operative Conditions	Current Work	Pivato et al. [26]	Chirizzi et al. [27]	Linillos-Pradillo et al. [29]	Setti et al. [25]
Investigated virus	SARS-CoV-2 and coronavirus 229E (HCoV-229E)	SARS-CoV-2	SARS-CoV-2	SARS-CoV-2	SARS-CoV-2
Sampling size	38 samples from 5 sites	44 samples from 10 sites	60 samples from 2 sites	6 × 3 = 18 samples from 1 site	34 samples from 2 sites
Positive samples	14 samples positive for coronavirus 229E; 7 samples positive for SARS-CoV-2	0 samples	0 samples	0 samples	20 samples
Location of sampling	Italy, Venice province (NE Italy)	Italy, Padua province (NE Italy)	Two Italian regions: Veneto (NE Italy) and Apulia (SE Italy)	Spain, Madrid	Italy, Bergamo Province (northern Italy)
Period of sampling	From 21 February to 8 March 2020 (16 days) and from 27 October to 25 November 2020 (29 days)	From 24 February to 9 March 2020 (14 days)	From 13 to 27 May 2020 (14 days)	From 4 to 22 May 2020 (18 days)	From 21 February to 13 March 2020 (21 days)
Typology of sampling point	Urban background site and marine traffic	Urban and rural background sites; traffic and industrial sites	Urban background site	Urban background site	Industrial site
Particulate size investigated	PM10 and PM2.5	PM10 and PM2.5	PM10	PM10, PM2.5, and PM1	PM10
Filter typology	Two typologies of filters were used: - Teflon fiber filters (90 mm Ø) - Quartz fiber filters (47 mm Ø)	Quartz fiber filters (47 mm Ø, Whatman QMA, GE Healthcare, USA)	Quartz fiber filters	Whatman quartz fiber filters (150 mm diameter and QMA quality)	Quartz fiber filters
Sampler typology	Two samplers were used: (1) Low-volume aerosol sampler (Skypost PM-TCR Tecora) equipped with a sequential sampler (Charlie) operating at a flow rate of 38.3 L/min for 24 h. (2) High-volume aerosol sampler (Techora/Echo PM Hi Vol) operating at a flow rate of 500 L/min for 8–24 h.	Two samplers were used: (1) Low-volume aerosol sampler (Skypost PM-TCR Tecora) equipped with a sequential sampler (Charlie) operating at a flow rate of 38.3 L/min for 24 h. It was used for the following sites: BO, TO, ES, PO, and SA. (2) Low-volume aerosol sampler (SWAM 5a Dual Channel Monitor-FAI Instruments) operating at a flow rate of 38.3 L/min for 24 h. It was used for the following sites: PD1, PD2, PD3, and SG.	Two samplers were used per site: - Veneto: Low-volume aerosol sampler (Skypost PM-TCR Tecora) equipped with a sequential sampler (Charlie) operating at a flow rate of 38.3 L/min for 48 h; and model 110 MOUDI cascade impactor with an average flow of 30 L/min for approximately 6 days. - Apulia: Low-volume aerosol sampler (SWAM 5a Dual Channel Monitor-FAI Instruments) operating at a flow rate of 38 L/min for 48 h and a rotating model 120 MOUDI-II™ cascade impactor operating at a flow rate of 30 L min^−1^ for approximately 6 days.	MCV high-volume samplers (30 m3 h^−1^ flow)	Low-volume gravimetric air sampler (38.3 L/min for 24 h)
Average air collected per sample	55.2 m^3^ for the low-volume aerosol sampler; 250 to 700 m^3^ for the high-volume aerosol sampler	55.2 m^3^	110 m^3^ or 250 m^3^	Not reported	55.2 m^3^
PM retention	The two typologies of filter have a similar efficiency (>99.95%) for particles with an aerodynamic diameter of 0.3 µm	>99.95% for particles with an aerodynamic diameter of 0.3 µm	Not reported		99.9%
Sampling procedure	EN 12341:2014 for the low-volume aerosol sampler	EN 12341:2014	Not reported	EN 12341:2014 with special ad hoc features (not reported)	EN 12341:2014
Meteorological conditions	Temperature, precipitation, and wind intensity	Temperature, irradiation, precipitation, and wind intensity	Temperature, relative humidity, and precipitation	Temperature, relative humidity, precipitation, wind intensity, wind direction, atmospheric pressure, and irradiance	Temperature, relative humidity, and irradiance
Solid-phase extraction	NucliSens extraction system, (bioMerieux, France) and one-step PCR inhibitor removal kit (Zymo Research)	Quick-RNA™ fecal/soil microbe microprep kit (Zymo Research, USA)	Total RNA purification kit (Norgen Biotek Corp.)	Quick-RNA™ fecal/soil microbe microprep kit (Zymo Research, USA)	Quick-RNA™ fecal/soil microbe microprep kit (Zymo Research, USA)
Viral recovery	Mengovirus applied to the filter	Armored RNA applied to the liquid phase	Mengovirus applied to a liquid PBS filter sonication buffer	None	None
Internal positive control	Mengovirus	SARS-CoV-2 (E gene)-armored RNA (EVA, Marseille, France)	Not reported	CTR-HS purification control (part of the AnyGenes kit)	Not reported
Inhibition control	External inhibition control (in vitro synthetized Orf1b-nsp14 RNA)	SARS-CoV-2 (E gene)-armored RNA (EVA, Marseille, France)	None	None	None
RT-PCR reference protocol	[30]	[31]	[32]	[33]	[32]
RT-PCR oligos	Custom oligos (Eurofins Genomics)	Custom oligos (Thermofisher)	Diatheva commercial kit	AnyGenes commercial kit Efficient 2019-nCOV detection kit (Cat#19nCoVd-100)	Not reported
RT-PCR molecular targets	Orf1b-14nsp	Genes N and Orf1b-14nsp	Genes RdRp and E	N1 and N2	Genes E, RdRP, and N
Limit of detection	0.41 g.c./μL (LOD50)	2.5 g.c./μL	10 g.c./μL	Not reported	Not reported
Detection threshold	0.1 g.c. m^−3^	1.2 g.c. m^−3^	<0.8 g.c. m^−3^	Not reported	1.5 g.c. m^−3^ *

Note: * The detection threshold for the method of Setti et al. [25] was calculated assuming 2 g.c./μL as the LOD for the molecular assay [32], and the RNA extraction protocol strictly followed the kit manufacturer’s instructions, with a 90% purification efficiency.

**Table 2:** Description of the sample sites.

Code	Place	Geographical Coordinates	Type of Station
LI	Via Lissa, Mestre (VE)	Lat. 45°29′11″; Long. 12°13′21″	Urban background, mainland
RN	Rio Novo (VE)	Lat. 45°26′08″; Long. 12°19′23″	Marine traffic, island; the site is located in the center of Venezia and used to monitor small boat traffic
SF	Sacca Fisola (VE)	Lat. 45°25′42″; Long. 12°18′47″	Urban background, island
PB	Parco Bissuola (VE)	Lat. 45°29’ 58″; Long. 12°15′40″	Urban background, mainland
SD	Via Turati, San Donà (VE)	Lat. 45°37′45″; Long. 12°35′25″	Urban background, mainland

## Data Availability

Not applicable.

## Supplementary Materials

The following supporting information can be downloaded at: https://www.mdpi.com/article/10.3390/ijerph19159462/s1, Supplementary Material S1. Information on PM samples: Sample code; collection date; sampling site (LI: Via Lissa; PB: Parco Bissuola; SF: Sacca Fisola; RN: Rio Novo; SD: San Donà); meteorological conditions; average air collected per sample; filter: Q = quartz fiber filters (47 mm Ø); T = teflon fiber filters (90 mm Ø); PM typology (PM2.5 or PM10); PM concentration. Supplementary Material S2. Results of virus detection in PM samples.
