# Supplementary material for: SARS-CoV-2 in Atmospheric Particulate Matter: An Experimental Survey in the Province of Venice in Northern Italy
Source: Int J Environ Res Public Health. 2022 Aug 2;19(15):9462. doi: 10.3390/ijerph19159462 (PMC9367860; doi:10.3390/ijerph19159462)
Supplement: Supplementary file 1 [file ijerph-19-09462-s001.zip › ijerph-1765149-supplementary.pdf]

Supplementary Material S1. Information on PM samples: Sample code; collection date; sampling site (LI: Via Lissa; PB: Parco Bissuola; SF: Sacca Fisola; RN: Rio Novo; SD: San Donà); meteorological conditions; average air collected per sample; filter: Q = quartz fiber filters (47 mm Ø); T = teflon fiber filters (90 mm Ø); PM typology (PM2.5 or PM10); PM concentration.

ID	Sample Code	Date	Sampling site	Meteorological Conditions (1)	Air volume / sample (m^3)	Filter	PM Typology	PM ($\mu g/m^3$)
1	734605	21/02/2020	SD	P = 0.0 mm; I = 0.9 m/s; T = 6°C	54.8	Q	PM10	33
2	734606	22/02/2020	SD	P = 0.0 mm; I = 1.3 m/s; T = 7.9°C	54.8	Q	PM10	34
3	734608	24/02/2020	SD	P = 0.0 mm; I = 1.2 m/s; T = 8.4°C	54.8	Q	PM10	86
4	735673	25/02/2020	PB	P = 0.0 mm; I = 0.7 m/s; T = 9.2°C	54.6	Q	PM2.5	68
5	735674	26/02/2020	PB	P = 0.0 mm; I = 1.7 m/s; T = 8.8°C	54.6	Q	PM2.5	24
6	735675	27/02/2020	PB	P = 0.0 mm I = 1.5 m/s; T = 7.5°C	54.6	Q	PM2.5	8
7	735676	28/02/2020	PB	P = 0.0 mm; I = 1.4 m/s; T = 7.9°C	54.6	Q	PM2.5	13
8	737619	28/02/2020	SF	P = 0.0 mm; I = 1.4 m/s; T = 10.6°C	54.8	Q	PM10	14
9	736654	04/03/2020	PB	P = 0.0 mm; I = 1.2 m/s; T = 8.3°C	54.6	Q	PM2.5	10
10	736567	05/03/2020	SD	P = 6.4 mm; I = 0.9 m/s; T = 6.9°C	54.8	Q	PM10	20
11	736655	05/03/2020	PB	P = 6.4 mm; I = 0.9 m/s; T = 6.9°C	54.6	Q	PM2.5	19
12	736567	06/03/2020	SD	P = 13.2 mm; I = 2.2 m/s; T = 7.8°C	54.8	Q	PM10	12
13	736656	06/03/2020	PB	P = 13.2 mm; I = 2.2 m/s; T = 7.8°C	54.6	Q	PM2.5	11
14	736657	07/03/2020	PB	P = 0.0 mm; I = 1.0 m/s; T = 10.1°C	54.6	Q	PM2.5	14
15	736568	08/03/2020	SD	P = 0.0 mm; I = 1.2 m/s; T = 7.7°C	54.8	Q	PM10	14
16	736658	09/03/2020	PB	P = 0.0 mm; I = 0.7 m/s; T = 8.2°C	54.6	Q	PM2.5	8
17	774656	27/10/2020	RN	P = 9.4 mm; I = 1.1 m/s; T = 14.9°C	23.8	Q	PM2.5	11
18	774657	28/10/2020	RN	P = 0.0 mm; I = 0.8 m/s; T = 13.5°C	23.8	Q	PM2.5	22
19	774659	29/10/2020	RN	P = 0.0 mm; I = 0.5 m/s; T = 12.8°C	23.8	Q	PM2.5	31
20	774659	30/10/2020	RN	P = 0.0 mm; I = 0.5 m/s; T = 13°C	23.8	Q	PM2.5	39
21	774661	31/10/2020	RN	P = 0.0 mm; I = 0.7 m/s; T = 11.3°C	23.8	Q	PM2.5	51
22	774662	01/11/2020	RN	P = 0.2 mm; I = 0.6 m/s; T = 10.1°C	23.8	Q	PM2.5	61
23	774663	02/11/2020	RN	P = 0.0 mm; I = 0.6 m/s; T = 12°C	23.8	Q	PM2.5	49
24	774664	03/11/2020	RN	P = 0.0 mm; I = 0.3 m/s; T = 13.2°C	23.8	Q	PM2.5	62
25	774665	04/11/2020	RN	P = 0.0 mm; I = 0.9 m/s; T = 13.6°C	23.8	Q	PM2.5	34
26	774666	05/11/2020	RN	P = 0.0 mm; I = 2.3 m/s; T = 14.8°C	23.8	Q	PM2.5	8

ID	Sample Code	Date	Sampling site	Meteorological Conditions (1)	Air volume / sample (m^3)	Filter	PM Typology	PM (μg/m^3)
27	774667	06/11/2020	RN	P = 0.0 mm; I = 1.6 m/s; T = 13.7°C	23.8	Q	PM2.5	14
28	HV_01	07/11/2020	LI	P = 0.0 mm; I = 0.3 m/s; T = 10.8°C	524	T	PM10	41 (2)
29	774668	07/11/2020	RN	P = 0.0 mm; I = 0.8 m/s; T = 13.6°C	23.8	Q	PM2.5	35
30	774669	08/11/2020	RN	P = 0.0 mm; I = 0.8 m/s; T = 11.9°C	23.8	Q	PM2.5	42
31	HV_02	09/11/2020	LI	P = 0.0 mm; I = 0.2 m/s; T = 10°C	264	T	PM10	66 (2)
32	774670	09/11/2020	RN	P = 0.0 mm; I = 0.7 m/s; T = 12.3°C	23.8	Q	PM2.5	47
33	774671	10/11/2020	RN	P = 0.0 mm; I = 1.3 m/s; T = 12.2°C	23.8	Q	PM2.5	22
34	774672	11/11/2020	RN	P = 0.2 mm; I = 2.5 m/s; T = 10.2°C	23.8	Q	PM2.5	15
35	HV_03	12/11/2020	LI	P = 0.2 mm; I = 0.4 m/s; T = 8.7°C	717	T	PM10	35 (2)
36	HV_04	17/11/2020	LI	P = 0.0 mm; I = 0.6 m/s; T = 9.2°C	534	T	PM10	32 (3)
37	HV_05	18/11/2020	LI	P = 0.5 mm; I = 0.0 m/s; T = 9.1°C	273	T	PM10	33 (2)
38	HV_06	25/11/2020	LI	P = 0.0 mm; I = UN m/s; T = 3.9°C	248	T	PM10	59 (2)

Notes:
(1) Meteorological conditions were registered by the closest monitoring stations: "Venezia - Istituto Cavanis" (Lat. 45° 25' 48''; Long. 12° 19' 41'') and "Mogliano Veneto" (Lat. 45° 34' 51''; Long. 12° 18' 28''). Daily readings of precipitation (P) in mm, mean wind intensity (I) in m/s and mean temperature (T) in Celsius degrees are reported.
(2) PM concentration is the mean value registered by two close-by monitoring stations (i.e. the "Parco Bissuola" urban background station and the "via Tagliamento" traffic station), since teflon filters were processed for virus detection only.

Supplementary Material S2. Results of virus detection in PM samples.

				hCoV-229E		SARS-CoV-2			
ID	Sample Code	Processed Filter Fraction	Recovery (%)	Qualitative detection	Positive replicates	gc/µL	gc/filter	gc/m³	Detected mutations
1	734605	1.00	0.9%	+	0/4	<LOD	-	-	-
2	734606	1.00	0.8%	+	0/4	<LOD	-	-	-
3	734608	1.00	1.3%	-	0/4	<LOD	-	-	-
4	735673	1.00	0.5%	-	0/4	<LOD	-	-	-
5	735674	1.00	100.0%	-	0/4	<LOD	-	-	-
6	735675	1.00	100.0%	-	0/4	<LOD	-	-	-
7	735676	1.00	0.7%	-	2/4	0.34	3.4E+01	0.6	-
8	737619	1.00	4.2%	-	0/4	<LOD	-	-	-
9	736654	1.00	1.5%	-	1/4	0.89	8.9E+01	1.6	-
10	736567	1.00	0.7%	-	1/4	0.92	9.2E+01	1.7	-
11	736655	1.00	1.3%	-	0/4	<LOD	-	-	-
12	736567	1.00	1.0%	-	1/4	0.93	9.3E+01	1.7	-
13	736656	1.00	1.5%	+	0/4	<LOD	-	-	n.d.
14	736657	1.00	1.1%	-	1/4	0.97	9.7E+01	1.8	none
15	736568	1.00	0.5%	-	0/4	<LOD	-	-	-
16	736658	1.00	1.0%	-	0/4	<LOD	-	-	-
17	774656	1.00	0.6%	-	3/4	0.58	5.8E+01	2.4	-
18	774657	1.00	1.0%	+	1/4	0.43	4.3E+01	1.8	none
19	774659	1.00	0.4%	-	2/4	1.00	1.0E+02	4.2	none
20	774659	1.00	0.1%	+	2/4	0.64	6.4E+01	2.7	-
21	774661	1.00	0.4%	+	1/4	0.46	4.6E+01	1.9	-
22	774662	1.00	0.5%	+	0/4	<LOD	-	-	-
23	774663	1.00	0.4%	-	1/4	0.90	9.0E+01	3.8	-
24	774664	1.00	0.5%	+	1/4	0.44	4.4E+01	1.8	none
25	774665	1.00	0.3%	-	2/4	0.73	7.3E+01	3.1	none
26	774666	1.00	0.3%	+	2/4	0.66	6.6E+01	2.8	-
27	774667	1.00	0.5%	-	1/4	0.42	4.2E+01	1.8	none
28	HV_01	0.25	0.6%	-	2/4	0.32	1.3E+02	0.2	-
29	774668	1.00	0.6%	-	2/4	0.59	5.9E+01	2.5	none
30	774669	1.00	0.5%	+	1/4	0.48	4.8E+01	2.0	-
31	HV_02	0.25	0.4%	-	2/4	1.05	4.2E+02	1.6	-

				hCoV-229E		SARS-CoV-2			
**ID**	**Sample Code**	**Processed Filter Fraction**	**Recovery (%)**	**Qualitative detection**	**Positive replicates**	**gc/μL**	**gc/filter**	**gc/m³**	**Detected mutations**
32	774670	1.00	0.2%	-	1/4	0.88	8.8E+01	3.7	F59L,T114I
33	774671	1.00	0.8%	-	0/4	<LOD	-	-	-
34	774672	1.00	0.5%	+	1/4	0.88	8.8E+01	3.7	-
35	HV_03	0.25	1.0%	+	1/4	0.47	1.9E+02	0.3	-
36	HV_04	0.25	100.0%	+	0/4	<LOD	-	-	-
37	HV_05	0.25	0.9%	+	4/4	0.37	1.5E+02	0.5	-
38	HV_06	0.25	0.5%	-	3/4	0.33	1.3E+02	0.5	-

Note: positive (+), negative (-); genome copies (gc); Limit of Detection (LOD) i.e. 0.41 g.c./L; not done (n.d.).
